# Non-technical skills evaluation in the critical care air ambulance environment: introduction of an adapted rating instrument - an observational study

**DOI:** 10.1186/s13049-016-0216-5

**Published:** 2016-03-08

**Authors:** Julia A. Myers, David M. C. Powell, Alex Psirides, Karyn Hathaway, Sarah Aldington, Michael F. Haney

**Affiliations:** Occupational and Aviation Medicine Unit, University of Otago Wellington, Newtown, Wellington, 6021 New Zealand; Department of Intensive Care Medicine, Wellington Regional Hospital, Wellington, New Zealand; University of Otago Wellington, Newtown, Wellington, 6021 New Zealand; Intensive Care Unit, Wellington Regional Hospital, Wellington, New Zealand; University of Otago Wellington, Newtown, Wellington, 6021 New Zealand; Department of Emergency Medicine, Wellington Regional Hospital, Wellington, New Zealand; Anesthesia and Intensive Care Medicine, Umeå University Medical Faculty, Umeå Sweden; Occupational and Aviation Medicine Unit, University of Otago Wellington, Newtown, Wellington, 6021 New Zealand

**Keywords:** Non-technical skills, Air ambulance, Intensive care, Patient transport, Clinical training

## Abstract

**Background:**

In the isolated and dynamic health-care setting of critical care air ambulance transport, the quality of clinical care is strongly influenced by non-technical skills such as anticipating, recognising and understanding, decision making, and teamwork. However there are no published reports identifying or applying a non-technical skills framework specific to an intensive care air ambulance setting. The objective of this study was to adapt and evaluate a non-technical skills rating framework for the air ambulance clinical environment.

**Methods:**

In the first phase of the project the anaesthetists’ non-technical skills (ANTS) framework was adapted to the air ambulance setting, using data collected directly from clinician groups, published literature, and field observation. In the second phase experienced and inexperienced inter-hospital transport clinicians completed a simulated critical care air transport scenario, and their non-technical skills performance was independently rated by two blinded assessors. Observed and self-rated general clinical performance ratings were also collected. Rank-based statistical tests were used to examine differences in the performance of experienced and inexperienced clinicians, and relationships between different assessment approaches and assessors.

**Results:**

The framework developed during phase one was referred to as an aeromedical non-technical skills framework, or AeroNOTS. During phase two 16 physicians from speciality training programmes in intensive care, emergency medicine and anaesthesia took part in the clinical simulation study. Clinicians with inter-hospital transport experience performed more highly than those without experience, according to both AeroNOTS non-technical skills ratings (*p* = 0.001) and general performance ratings (*p* = 0.003). Self-ratings did not distinguish experienced from inexperienced transport clinicians (*p* = 0.32) and were not strongly associated with either observed general performance (*r*_s_ = 0.4, *p* = 0.11) or observed non-technical skills performance (*r*_s_ = 0.4, *p* = 0.1).

**Discussion:**

This study describes a framework which characterises the non-technical skills required by critical care air ambulance clinicians, and distinguishes higher and lower levels of performance.

**Conclusion:**

The AeroNOTS framework could be used to facilitate education and training in non-technical skills for air ambulance clinicians, and further evaluation of this rating system is merited.

**Electronic supplementary material:**

The online version of this article (doi:10.1186/s13049-016-0216-5) contains supplementary material, which is available to authorized users.

## Background

In health care, preventing errors and avoidable adverse events for patients (patient safety) is paramount. High quality clinical performance requires adequate knowledge and technical ability, but also relies on non-technical skills such as the ability to adapt to a rapidly changing clinical situation and to function as part of a team [1, 2]. Non-technical skills can be defined as “the cognitive, social and personal resource skills that complement technical skills and contribute to safe and efficient task performance” [3]. Even though a high degree of technical expertise in important, this alone is not enough to prevent clinician error or mishap. Non-technical skills are more likely, compared to technical skills, to be sensitive to individual human factors such as fatigue and stress [3]. High risk industries with low tolerance for error (such as aviation and the nuclear power industry) were early to recognise the importance of non-technical skills for safety; these industries developed rating frameworks to evaluate crew performance based on observable behaviours [4, 5]. This approach has also been implemented in high-risk health care domains, where behavioural marker systems are increasingly utilised as part of training or assessment of clinical competence [6, 7].

The air ambulance environment is a complex and dynamic health-care setting, where clinicians work with limited resources to provide very advanced levels of care [8–10]. Highly specialised care is centralised in many modern health care systems and critically ill patients are routinely transported large distances to tertiary hospitals to provide timely access to intensive care [11, 12]. Air ambulance transport teams tend to be small and comprise different professional categories such as nurses, emergency medical technicians, and physicians. Challenges for maintaining patient safety in an aviation environment include managing sometimes acutely life-threatening and rapidly evolving medical issues without the support and facilities available in a hospital environment [13, 14]. High noise levels in the cabin may preclude traditional options for clinical surveillance such as auscultation or audible alarms, and make communication challenging. Clinicians cannot always access additional assistance, resources, or expertise, should problems arise or clinical status change while the patient is in transit. In this context, while good technical expertise is certainly required, it may be non-technical factors such as how well clinicians have planned and anticipated, or how quickly they recognise, understand, and make decisions, that most strongly influence eventual outcome. Well-designed training for air ambulance clinicians should aim to prepare them for the recognised risks to patient care during all phases of transfer, and an assessment framework based on non-technical skills would clearly have a high degree of relevance for this purpose. However there are no published reports identifying or applying a non-technical skills framework specific to an intensive care air ambulance setting.

A number of non-technical skills rating frameworks have been developed for health-care domains closely related to the air ambulance setting, including emergency care [6, 15], critical care [16], and anaesthesia [17–19]. Each of these frameworks is broadly similar, reflecting the generic nature of non-technical skills’ categories such as situational awareness, decision making, and teamwork [3], however specific skill elements and behavioural descriptors vary according to the clinical requirements of the specific domain [20]. An existing behavioural rating framework can be adapted to another clinical setting using data gathered directly from the new setting [7]. In the aeromedical setting, the well-established Anaesthetists’ Non-Technical Skills (ANTS) framework [17] is suitable for this purpose [21, 22]. The ANTS system provides a framework for describing the individual non-technical skills of clinicians as well as a tool to guide their assessment within the clinical workplace for anaesthesia [23]. The overall goal of this study was to assess a newly adapted non-technical skills rating system based on the ANTS system but modified for the air ambulance clinical environment. Our hypothesis was that a non-technical skills framework adapted to a critical care air ambulance environment could discriminate between stronger and weaker non-technical skills performances. We aimed to test this with a volunteer cohort of lesser and more experienced intensive care physicians in a challenging air ambulance transfer simulation, where non-technical skills assessors were blinded to clinician experience.

## Method

The project was undertaken in two phases. First, a non-technical skills framework was adapted to the critical care air ambulance setting using the ANTS system as the foundation. The adapted framework was referred to as an aeromedical non-technical skills framework, or AeroNOTS. In the second phase the adapted AeroNOTS framework was utilised to evaluate the non-technical skills observed in clinicians working in simulated inter-hospital transport scenarios.

### Adaptation of a non-technical skills framework to the critical care air ambulance setting

**Fig. 1:**
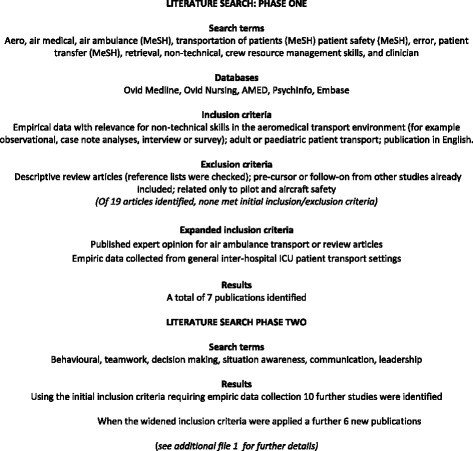
Literature search to identify tasks and non-technical skills associated with safe and effective clinical practice in the air ambulance clinical environment

Using the ANTS framework as the starting point, a selected and broadly representative group of experienced critical care transport and aviation medicine clinicians agreed definitions for each non-technical skills category and element as they pertained to critical care air transfer. They also provided suggestions for good and poor clinical behaviours. This work was initiated with a scoping review of the literature to identify skill elements and observable behaviours essential for the air ambulance environment, and to understand the range and nature of existing evidence. A search utilising online databases (Ovid Medline, Ovid Nursing, AMED, PsychInfo and Embase) was undertaken in two stages. The following search terms and all derivatives were used for the initial search: aero, air medical, air ambulance, transportation of patients, patient safety, error, patient transfer, retrieval, non-technical, crew resource management skills, clinicians. In the second stage of the search the ANTS tool and other derivatives were used as the basis for describing specific non-technical skills categories, which were then added as the following search terms: behaviour, teamwork, decision making, situation awareness, communication, leadership. There were no date restrictions and the search was conducted in March 2014 (Fig. 1) [Additional file 1]. Four focus group interviews were also undertaken, three with experienced New Zealand and Australian-based air ambulance clinicians (physicians, flight nurses, paramedics) and one with a group from an international post-graduate aeromedical education programme. Open ended questions were used to facilitate discussion concerning the essential tasks undertaken from beginning to end of a patient transport, and examples of the relevant good or poor ‘observable’ behaviours associated with those tasks. Behaviours and tasks arising out of the focus group data and literature were integrated with the developing AeroNOTS prototype to complete and inform the behaviour descriptions.

**Fig. 2:**
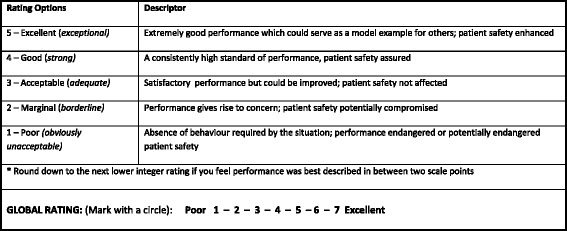
Rating scale descriptors for aeromedical non-technical skills performance

A content evaluation survey was undertaken using a purposive sampling method and Qualtrics electronic survey software (version 9340538, Copyright © 2015 Qualtrics., Provo, UT, USA). Clinicians from a range of critical care flight services rated the importance of positive behaviours from the prototype AeroNOTS framework and suggested key skills or behaviours they believed had been missed. The services were selected via contact with our Aviation Medicine teaching section including air ambulance organisations associated with previous students and current teachers in our international programme. This included university hospital-based air ambulance services as well as larger and smaller private air ambulance organisations in the United Kingdom, Australia and New Zealand. Any behaviour not rated by at least 75 % of respondents as either “very important” or “essential” was considered to potentially lack content validity [24] and was therefore revised. All free-text comments were reviewed to inform behavioural descriptor modifications and confirm they could be coded to an existing skills element. The prototype framework was also field tested in two critical care flight services in New Zealand and Sweden. Transport missions were observed from start to finish noting essential tasks observed or required but not covered by the framework, elements missing from or superfluous to the four main categories, and behavioural descriptions that may have needed modifying. A change was made from the ANTS scale to introduce a five-point scale for each element or category and a seven-point global rating (Fig. 2), following suggestions that the four point ANTS rating scale may lack sensitivity for measuring changes in performance [25] and that an overall non-technical skills scale may also be a useful addition [18, 26].

### Evaluation of non-technical skills using clinical simulation: study setting and participants

The observational study took place in the simulation suite of a New Zealand tertiary hospital; it was timed to take place around a training placement changeover with the aim of recruiting a convenience sample of minimally experienced intensive care transport physicians. Experienced intensive care transport physicians were also recruited and assessed. Prior to the simulations, the intensive care unit (ICU) flight service medical director categorised all the participants as either ‘experienced’ or ‘inexperienced’ in ICU inter-hospital transport. All participants provided informed consent and completed an enrolment questionnaire which included details of training, transport and simulation experience.

### Simulation scenario

Following orientation to the simulator all clinicians completed a critical care inter-hospital transport scenario with a highly experienced flight nurse serving as a ‘confederate’ and standardised team member. The scenario took approximately 20 minutes, with an initial phase set in a high-fidelity regional emergency department where the transport physician and flight nurse took over care of a ventilated patient requiring air ambulance transfer to a tertiary hospital ICU in another city. The second phase was set in a low fidelity helicopter fuselage with actual transport equipment (stretcher, ventilator, monitors) and comparable space restrictions, but no aircraft noise or vibration. In the scenario the patient’s condition deteriorated rapidly, and a life-saving intervention was required [Additional file 2]. Following the scenario an observing ICU consultant facilitated a de-brief, which was not recorded.

### Assessment of non-technical skills

Clinicians were informed that purpose of the study was to evaluate methods for assessing clinical performance. They were not specifically told that the key focus for that assessment was on non-technical skills. As recommended when undertaking formal assessment of non-technical skills performance, each skill element was initially rated separately, then final ratings were made at the level of the four main skill categories of task management, team working, situation awareness and decision making [27]. Since communication is required to demonstrate skill elements across all categories there was no specific category for communication in the AeroNOTS system, as with the ANTS system [17]. Possible scores for each skill category and element ranged between 1 and 5, where a rating of ‘5’ was “Excellent – extremely good performance which could serve as a model example for others; patient safety enhanced”, down to ‘1’ which was “Poor - absence of behaviour required by the situation; performance endangered or potentially endangered patient safety” (Fig. 2). Category scores were analysed separately giving a score for each category of between 1 and 5, and then added to give a single summed score (providing an overall non-technical skills score between 4 and 20) [25, 28]. Half marks on the scale were not permitted; assessors were instructed to score at the lower level if they felt the performance fell between two levels on the scale [Additional file 3].

The assessments were carried out independently by two observers who viewed video recordings of the scenarios; assessors were blinded to the experience level of the participants.

### Assessment of general clinical performance

**Fig. 3:**
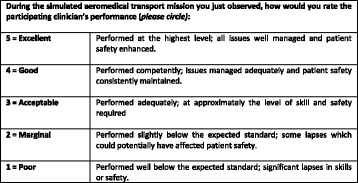
Rating scale descriptors for general clinical performance

Immediately after the simulation (prior to the debrief) one of the investigators, who was also playing the role of the confederate flight nurse, rated the general clinical performance of each participant on a five point scale ranging from a highest score of “5 = Excellent - performed at the highest level; all issues well managed and patient safety enhanced”, down to “1 = Poor - performed well below the expected standard; significant lapses in skills or safety” (Fig. 3). Participants rated their own clinical performance on the same five point scale slightly re-worded to reflect self-rating, rather than observed rating of others.

### Statistical analysis

The AeroNOTS scores from two assessors for each participant were averaged for further analysis within participant groups. *A priori* assumptions were that non-technical skills ratings for clinicians more experienced in air transports would be higher than for less experienced clinicians, and that general clinical performance levels would correlate with non-technical skills performance levels. Statistical analysis was undertaken using SPSS software (IBM SPSS Statistics for Windows, Version 22.0. Armonk, New York). Demographic variables were compared using t-tests or Fisher exact tests. AeroNOTS ratings scores (summed total scores between 4 and 20, and individual category scores between 1 and 5) were treated as ordinal data and analysed using rank-based methods. These included Mann–Whitney U to test for differences in performance between groups, Spearman’s rank correlation to examine relationships between different assessment approaches and assessors, and Wilcoxon signed-rank to examine individual scoring from the two assessors. Spearman’s rank correlation was also used to test for association between the immediate general rating of clinical performance compared to non-technical skills rating. The level of statistical significance was set at *p* < 0.05.

### Ethical approval

Ethical approval was provided by the University of Otago (Health) Human Ethics Committee, New Zealand (HD12/233 and HD14/44). All clinicians who participated in the clinical simulation study provided signed informed consent.

## Results

### Non-technical skills framework adapted for the critical care air ambulance setting

**Fig. 4:**
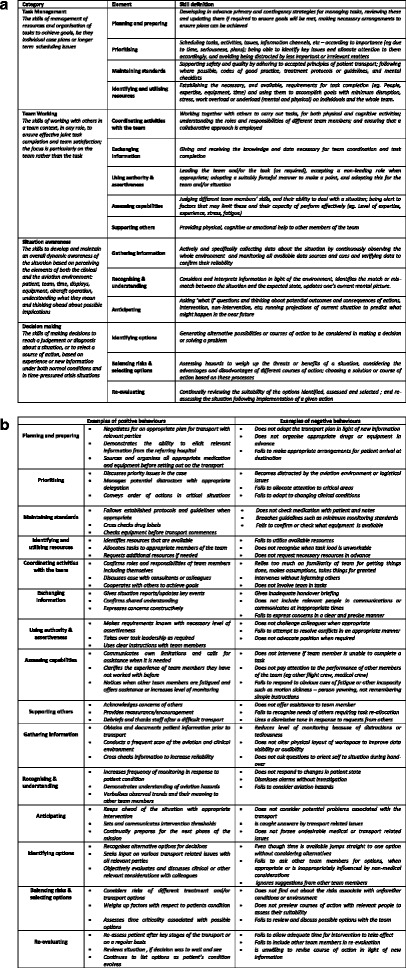
**a** Aeromedical non-technical skill categories and elements; Definitions of skill categories and elements in the aeromedical non-technical skills framework **b**; Illustrative behaviours for aeromedical non-technical skills; Examples of positive and negative illustrative behaviours for non-technical skill elements in the aeromedical non-technical skills framework

The final prototype of the AeroNOTS framework was produced from expert working group, literature review, focus group, clinician survey, and field testing data (Fig. 4a and b). The content evaluation survey was fully completed by 38 clinicians; 20 flight nurses, 12 specialist transport physicians and six paramedics, who had a median aeromedical transport experience of 8 (IQR 4 – 13) years. Based on responses there were no skills or behaviours added, though five existing behaviour descriptors were revised [Additional file 4]. Field testers expressed a preference for being able to distinguish between good and exemplary performance preferring a five-point performance rating scale over a four-point scale, and they confirmed that a “not applicable” category was required as some transport missions either do not require all skill elements or they just could not be observed.

### Evaluation of non-technical skills using clinical simulation

A total of 16 physicians from speciality training programmes in intensive care, emergency medicine and anaesthesia took part in the clinical simulation study (Table 1). Eight of the participants practiced at a senior trainee level or higher and were categorised as ‘experienced’, having undertaken a median of 45 (IQR 25 – 51.5) previous inter-hospital patient transports. The other eight practiced at a senior trainee level or lower and were categorised as ‘inexperienced’ in patient inter-hospital transport (median 0.5, IQR 0 – 4.5). The mean age for the experienced group was 36.1 (SD 5.6) years and 50 % of them were male. The inexperienced group were younger (*p* = 0.009) with a mean age of 29.8 (SD 2.1) years, and all were male. There was no difference in any other baseline characteristics including previous experience of simulator training, the number of hours they had worked or slept in the 24 hours prior to the simulation, and their fatigue level at the time of simulation.

**Table 1:** Clinical simulation study: baseline characteristics of participants

Characteristic	Experienced group	Inexperienced group	*P* value
Age (mean ± SD)	36.1 ± 5.6	29.8 ± 2.1	0.009
Male gender, *n* (%)	4 (50 %)	8 (100 %)	0.08
Training level, *n* (%)			
Consultant	2 (25 %)	0 (0 %)	
Registrar (senior)	6 (75 %)	2 (25 %)	
Registrar (junior)	0	5 (62.5 %)	
Intern	0	1 (12.5 %)	
Specialty training programme, *n* (%)			
ICU and Anaesthetics	1 (12.5 %)	2 (25 %)	
Anaesthetics	0 (0 %)	3 (37.5 %)	
Critical and Intensive Care Medicine	3 (37.5 %)	0 (0 %)	
Emergency Medicine	3 (37.5 %)	2 (25 %)	
General Medicine	1 (12.5 %)	1 (12.5 %)	
Number of previous inter-hospital patient transports, *median* (IQR)	45 (25 – 51.5)	0.5 (0 – 4.5)	0.001
Number of previous simulations, *median* (IQR)	15 (2.25 – 20)	5 (5 – 16)	0.57
Sleep in 24 hrs pre-scenario, *median hours* (IQR)	7.0 (6.6 – 7.9)	7.0 (7–8)	0.80
Work in 24 hrs pre-scenario, *median hours (*IQR)	8 (1 – 13.5)	6 (1.75 – 7.75)	0.51
Samn-Perelli Fatigue Score^a^, *median* (IQR)	3 (1.25 – 4.75)	2.5 (1.25 – 3.75)	0.57

^a^Samn-Perelli Fatigue checklist - possible scores between 1 and 7 where 1 is “fully alert wide awake” through to 7 which is “completely exhausted, unable to function effectively” *(Samn SW, Perelli LP. Estimating aircrew fatigue: a technique with implications to airlift operations. Brooks AFB,TX: USAF School of Aerospace Medicine; 1982. Technical Report No. SAM-TR- 82–21.)*

### Assessment of non-technical skills

Performance ratings from the two assessors were similar, with a high degree of correlation (*r*_s_ = 0.65, *p* = 0.006) and no significant difference in overall scores (AeroNOTS summed scores, Wilcoxon signed-ranks test, *p* = 0.21). These results stayed consistent across the four individual skill categories (correlation coefficients ranging between *r*_s_ = 0.54 and 0.76, all significantly greater than 0 at *p* = 0.05). However for the category of ‘task management’ the scores from one assessor rated higher than the other (Wilcoxon signed-ranks test, *p* = 0.02). The averages of the 2 assessors’ scores were used for the analyses.

**Fig. 5:**
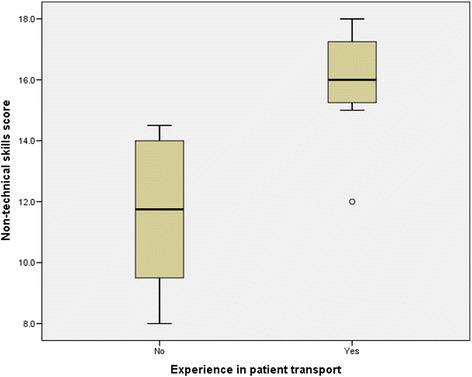
Non-technical skills ratings for experienced versus inexperienced intensive care transport clinicians

**Fig. 6:**
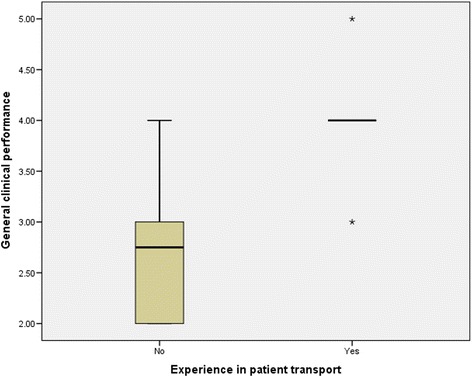
General clinical performance ratings for experienced versus inexperienced intensive care transport clinicians

**Fig. 7:**
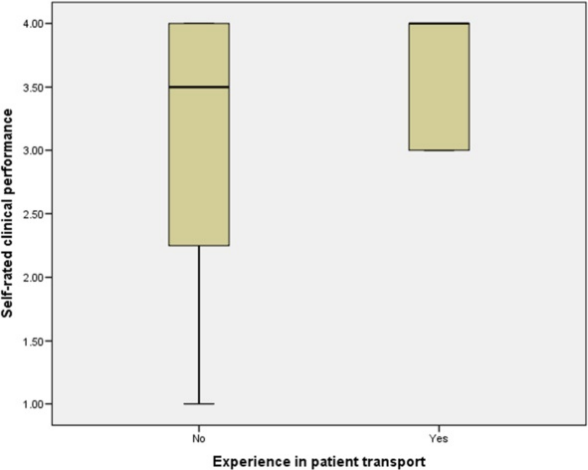
Self-rated clinical performance of experienced and inexperienced intensive care transport clinicians

Non-technical skills ratings for all participants (Table 2) showed that clinicians with experience in patient transfer had higher non-technical skills (AeroNOTS) scores than less experienced clinicians (Mann Whitney U, *p* = 0.001) (Fig. 5). The experienced clinicians also had higher general performance ratings than inexperienced clinicians (Mann Whitney U, *p* = 0.003) (Fig. 6). For all clinicians, ratings for non-technical skills were highly correlated with general performance ratings (r_s_ = 0.9, *p* = 0.001). Self-ratings of clinical performance did not discriminate in the same way as ‘observed’ performance measures, and the self-rated performance of experienced clinicians was no different to that of inexperienced clinicians (Mann Whitney U, *p* = 0.32) (Fig. 7). In addition, self-rated performance was not strongly associated with either observed general performance (*r*_s_ = 0.4, p = 0.11) or observed non-technical skills performance (*r*_s_ = 0.4, *p* = 0.1).

**Table 2:** Assessment scores: Non-technical skills and general clinical performance

Performance measure	Experienced group	Inexperienced group	*P* value*
Non-technical skills (AeroNOTS)^a^, *median (IQR)*	16 (15.125 – 17.125)	11.75 (8.75 – 14.25)	0.001
Task Management^b^, *median (IQR)*	3.75 (3.5 – 4.375)	2.5 (2.125 – 3)	0.001
Teamwork^b^ *, median (IQR)*	4 (3.625 – 4.5)	3 (2.5 – 3.5)	0.002
Situational Awareness^b^ *, median (IQR)*	3.75 (3.5 – 4.375)	3 (2.5 – 3.375)	0.03
Decision Making^b^ *, median (IQR)*	4 (3.625 – 4.375)	2.75 (2.125 – 3)	0.02
General clinical performance^c^ *, median (IQR)*	4 (4 – 4)	2.75 (2 – 3)	0.003
Self-rated clinical performance^c^ *, median (IQR)*	4 (3 – 4)	3.5 (2.125 – 4)	0.32

^a^Median Aeromedical Non-technical Skills rating (summed score, possible range between 4 and 20, higher scores represent a higher level of performance)

^b^Median non-technical skills category rating (possible range between 1 and 5, higher scores represent a higher level of performance)

^c^Overall clinical performance rating (self-rated or observed - possible range between 1 and 5, higher scores represent a higher level of performance)

*Mann-Whitey U, two-tailed test

## Discussion

This study describes the development and evaluation of a framework to assess non-technical skills in aeromedical transport. The framework discriminated between more and less experienced clinicians, based on their non-technical skill performance during simulated transfer of a critical patient. Both technical and non-technical skills are needed in tandem for good medical team performance and patient safety in a high risk medical environment, and both improve with good training [29–31]. Necessary elements for improving clinical performance include identifying specific skills directly relevant to performance quality, then measuring or assessing those skills in a standardised manner [32]. The results of this study indicate that by characterising the non-technical skills requirements for clinicians, and distinguishing higher and lower levels of non-technical performance, the prototype AeroNOTS framework could be used to facilitate good education and training in non-technical skills. Published standards (on which training curricula may be based) from Europe [33], New Zealand [34], and the US [35], all mandate crew resource management (CRM) training in areas such as decision making, communications processes, team building and maintenance, workload management, and situation awareness, but they include little specific detail to define these skills. The specific behaviours identified in the AeroNOTS instrument can facilitate identification of specific areas for individuals where further training might be beneficial.

In this study, ‘self-ratings’ of performance were not useful in distinguishing different levels of performance, with inexperienced clinicians tending to over-estimate their performance level*.* Limitations in clinicians’ ability to self-assess performance have been reported previously [36], but it is possible that our findings were partly a result of recruiting one group of inexperienced clinicians who lacked appropriate inter-hospital transport experience on which to base their self-assessments. It is also possible that self-ratings are more accurate at the extremes, such as when performance is significantly degraded [36]. Further examination of ‘self-rating’ is warranted in light of the fact that critical care air ambulance clinicians are particularly vulnerable to factors like fatigue [37], and risk management systems generally rely on clinicians ‘self-identifying’ if their performance is compromised [38].

There is a paucity of literature and no published skills taxonomy, so collecting additional data from the critical care air ambulance domain to adapt the well-established ANTS system was essential [7]. Anaesthesia is a medical speciality with a leading role in addressing patient safety and taking a human factors approach to training and safety [39], and while a behavioural rating system cannot simply be applied to another specialty area [27] non-technical skills are broadly generic [3]. As previous authors report significant overlap in the non-technical skills requirements of intensive care and anaesthesia [20], it was reasonable to expect similarity between the skills required of intensive care air ambulance clinicians and anaesthesia specialists. Both function in teams of variable professional makeup, and so require frameworks where the fundamental focus is on the non-technical skills of individual clinicians, but encompassing how they function as part of a team.

A measurement system suitable for evaluating the non-technical skills of air ambulance clinicians should provide a true (valid) and consistent (reliable) representation of those skills. Face and content validity for the AeroNOTS system were addressed during development by collecting data directly from the aeromedical transport domain (clinician experts and relevant literature). For example, based on literature an addition to the ‘using authority or assertiveness’ element of Teamwork was: “questions others regardless of seniority when they are unsure the right decision has been made”; and a negative behaviour for the ‘gathering information’ element of Situational Awareness: “does not alter layout of the workplace to improve data visibility or audibility” (ability to hear in aircraft is limited so clinicians need to be able to see monitors to make up for this). From focus groups a recurrent theme emerged that experienced air ambulance clinicians “plan for things to go wrong” and “plan for every eventuality”. They also develop strategies for potential vulnerable points in the transfer, such as communication strategies for working with unknown team members, being assertive in acquiring information when working in unfamiliar surroundings, and ensuring they are being listened to. Results from the clinical simulation study provided support for the construct validity of the AeroNOTS framework as a tool for assessing non-technical skills performance. In accordance with the *a priori* expectations we set, experienced clinicians received higher scores from blinded assessors than inexperienced clinicians, and AeroNOTS scores were strongly correlated with general performance scores.

One limitation in the study design was that while data from all air ambulance clinician groups were informative concerning the phase one AeroNOTS adaptation process, only physicians participated in the second phase clinical simulation study. It was not possible to recruit comparative groups containing inexperienced flight nurses or air ambulance paramedics locally. Ongoing evaluation should therefore include all clinician groups routinely involved in critical care transfer. Another potential limitation is that much of the initial adaptation work and evaluation for the AeroNOTS framework involved clinicians and services based in New Zealand or Australia and it is possible that roles and responsibilities of air ambulance clinicians, and therefore the required non-technical skills, are not the same in all countries [40]. We aimed to provide that international perspective through assessment of the literature, content evaluation survey and field testing, all of which provided support for validity. However more wide-spread evaluation may be required.

This study was not designed to test the reliability of the AeroNOTS rating system. Based on results from studies of non-technical skills in other domains, some variability between assessor ratings, such as in our findings, was not unexpected [16, 41, 42]. The assessors were trained in non-technical skills concepts and use of the rating system [23], but no attempt was made to calibrate their ratings before the study. This will require further examination if the AeroNOTS framework is to be used for formal assessment of clinical competence.

## Conclusion

An air ambulance non-technical skills framework derived from the ANTS tool is capable of distinguishing good and poor performers in a simulated inter-hospital transport setting. Scores can be highly correlated with observed general performance, and also with the general experience of the clinician. Our findings confirm that self-ratings are not useful for distinguishing between higher and lower levels of performance. This framework could be useful in identifying when specific non-technical factors are likely to break down in the air ambulance environment, and facilitating a more structured approach to training and assessment. The AeroNOTS rating system shows utility and applicability for a critical care air ambulance environment, and further evaluation of this framework is merited.

## Additional files

Additional file 1: Literature search results. (PDF 342 kb) Additional file 2: Simulation scenario details. (PDF 1453 kb) Additional file 3: Aeromedical non-technical skills assessment form and rating scale. (PDF 266 kb) Additional file 4: Behavioural descriptor modifications based on content evaluation survey. (PDF 259 kb)

## Abbreviations

AeroNOTS: Aeromedical non-technical skills
ANTS: Anaesthetists’ non-technical skills
ICU: Intensive Care Unit
CRM: Crew resource management
