# Supplementary material for: Non-technical skills evaluation in the critical care air ambulance environment: introduction of an adapted rating instrument - an observational study
Source: Scand J Trauma Resusc Emerg Med. 2016 Mar 8;24:24. doi: 10.1186/s13049-016-0216-5 (PMC4784461; doi:10.1186/s13049-016-0216-5)
Supplement: Additional file 1: — Literature search results. (PDF 342 kb) [file 13049_2016_216_MOESM1_ESM.pdf]

LITERATURE SEARCH: PHASE ONE

Search terms

Aero, air medical, air ambulance (MeSH), transportation of patients (MeSH) patient safety (MeSH), error, patient transfer (MeSH), retrieval, non-technical, crew resource management skills, and clinician

Databases

Ovid Medline, Ovid Nursing, AMED, PsychInfo, Embase

Inclusion criteria

Empirical data with relevance for non-technical skills in the aeromedical transport environment (for example observational, case note analyses, interview or survey); adult or paediatric patient transport; publication in English.

Exclusion criteria

Descriptive review articles (reference lists were checked); pre-cursor or follow-on from other studies already included; related only to pilot and aircraft safety

(Of 19 articles identified, none met initial inclusion/exclusion criteria)

Expanded inclusion criteria

Published expert opinion for air ambulance transport or review articles

Empiric data collected from general interhospital ICU patient transport settings

Results

A total of 7 publications identified [1-7]

LITERATURE SEARCH PHASE TWO

Search terms

Behavioural, teamwork, decision making, situation awareness, communication, leadership

Results

Using the initial inclusion criteria requiring empiric data collection 10 further studies were identified [8-17]

When the widened inclusion criteria were applied a further 6 new publications were identified [18-23]

1. Blanchet D. Crew resource management and EMS. How an aviation technique can help us achieve greater scene and transport safety. EMS World. 2010;39(11):24, 6.
2. Droogh JM, Kruger HL, Ligtenberg JJ, Zijlstra JG. Simulator-based crew resource management training for interhospital transfer of critically ill patients by a mobile ICU. Joint Commission Journal on Quality & Patient Safety. 2012;38(12):554-9.
3. Erler C, Edwards NE, Ritchey S, Pesut DJ, Sands L, Wu J. Perceived patient safety culture in a critical care transport program. Air Med J. 2013;32(4):208-15.
4. Gordon M, Darbyshire D, Baker P. Non-technical skills training to enhance patient safety: a systematic review. Medical education. 2012;46(11):1042-54.
5. Gryniuk J, National Flight Paramedics A. The role of the flight paramedic in air medical safety and crew resource management. Air Med J. 2003;22(4):12-4; quiz 5, 31.
6. Hearns S, Shirley PJ. Retrieval medicine: a review and guide for UK practitioners. Part 2: safety in patient retrieval systems. Emergency Medicine Journal. 2006;23(12):943-7.
7. Springer B. Taking your crew resource management temperature. Air Med J. 2005;24(3):120-2; discussion 3.
8. Dalto JD, Weir C, Thomas F. Analyzing communication errors in an air medical transport service. Air Med J. 2013;32(3):129-37. doi:10.1016/j.amj.2012.10.019.
9. Fisher J, Phillips E, Mather J. Does crew resource management training work? Air Med J. 2000;19(4):137-9.
10. Flabouris A, Runciman WB, Levings B. Incidents during out-of-hospital patient transportation. Anaesthesia & Intensive Care. 2006;34(2):228-36.
11. Gabram SG, Hodges J, Allen PT, Allen LW, Schwartz RJ, Jacobs LM. Personality types of flight crew members in a hospital-based helicopter program. Air Med J. 1994;13(1):13-7.
12. Jaynes CL, Cook P, Farmer R, Werman HA, White L. Assessing satisfaction and quality in the EMS/HEMS working relationship. Air Med J. 2013;32(6):338-42. doi:http://dx.doi.org/10.1016/j.amj.2013.05.007.
13. MacDonald RD, Banks BA, Morrison M. Epidemiology of adverse events in air medical transport. Academic emergency medicine : official journal of the Society for Academic Emergency Medicine. 2008;15(10):923-31. doi:10.1111/j.1553-2712.2008.00241.x.
14. Pugh D. A phenomenologic study of flight nurses' clinical decision-making in emergency situations. Air Med J. 2002;21(2):28-36. doi:http://dx.doi.org/10.1016/S1067-991X(02)70083-7.
15. Stohler S. High Performance Team Interaction in an Air Medical Program. Air Med J. 1998;17(3).
16. Topley D, Schmelz J, Henkenius-Kirschbaum J, Horvath K. Critical Care Nursing Expertise during Air Transport. Mil Med. 2003;168.
17. Vilensky D, MacDonald RD. Communication errors in dispatch of air medical transport. Prehosp Emerg Care. 2011;15(1). doi:10.3109/10903127.2010.519817.
18. Bigham B, Morrison L, Maher J, Brooks S, Bull E, Morrison M et al. Patient safety in Emergency Medical Services advancing and aligning the culture of patient safety in EMS: The Canadian Patient Safety Institute Contract No.: ISBN 978-1-926541-23-5.
19. Frakes MA. Forum: Teamwork the sum and the parts. Air Med J. 2011;30(4):187-91. doi:10.1016/j.amj.2011.06.001.
20. Jaynes CL, Werman HA, White LJ. A blueprint for critical care transport research. Air Med J. 2013;32(1):30-5. doi:http://dx.doi.org/10.1016/j.amj.2012.11.001.
21. Maynard MT, Marshall D, Dean MD. Crew resource management and teamwork training in health care: a review of the literature and recommendations for how to leverage such interventions to enhance patient safety. Adv. 2012;13:59-91.
22. Reimer AP, Moore SM. Flight nursing expertise: towards a middle-range theory. J Adv Nurs. 2010;66(5):1183-92. doi:10.1111/j.1365-2648.2010.05269.x.
23. Williams KA, Rose WD, Simon R. Teamwork in emergency medical services. Air Med J. 1999;18(4):149 - 53.
