# Supplementary material for: Non-technical skills evaluation in the critical care air ambulance environment: introduction of an adapted rating instrument - an observational study
Source: Scand J Trauma Resusc Emerg Med. 2016 Mar 8;24:24. doi: 10.1186/s13049-016-0216-5 (PMC4784461; doi:10.1186/s13049-016-0216-5)
Supplement: Additional file 2: — Simulation scenario details. (PDF 1453 kb) [file 13049_2016_216_MOESM2_ESM.pdf]

AEROMEDICAL SIMULATION SCENARIO

UNIVERSITY of OTAGO
Te Whare Wānanga o Otāgo
NEW ZEALAND

Intensive Care Services

Scenario: Rapid Atrial Fibrillation During Aeromedical Retrieval
Location: Aeromedical Simulator
Patient Name: Lily Blythe
Gender: Female
Age: 76 years
Weight: 64kg

Learning Objectives:
- *Assimilation of history, vital signs & equipment settings in a timely fashion*
- *Recognise deterioration in a critically unwell patient*
- *Respond appropriately with an escalated intervention*
- *Efficient team work with appropriate delegation of workload*
- *Clear communication with other team members*

Introduction:

The retrieval team consists of two team members, a doctor (D) & a flight nurse (N). Unknown to D, N is aware of the scenario & may be asked to provide clinical prompts to elicit information from D. The scenario is set across two consecutive phases designed to allow assessment of the different components of the challenges faced during aeromedical retrieval. The two phases of the simulation are:

Phase 1: set in the resuscitation bay of an emergency department of a small hospital with an intubated mannequin on a hospital trolley attached to a ventilator & monitor. This phase is designed to assess the ability of the retrieval team to obtain relevant medical history in a timely fashion, review blood results & radiology, & examine the mannequin. If not volunteered by D, N will prompt to elicit a plan for retrieval. Phase 1 will end when the plan has been discussed to the satisfaction of both parties. The scenario should then progress immediately to phase 2.

Phase 2: set in the fuselage of an aeromedical retrieval plane at cruise altitude with an intubated mannequin strapped to an aeromedical transfer stretcher & connected to a portable monitor & ventilator. D & N are seated & must communicate through aviation headsets. This phase will form the bulk of the simulation.

Environment Set-up:

Phase 1 (set in the resuscitation bay of the Emergency Department of a small hospital):
- intubated female mannequin with #8.0 Portex endotracheal tube in-situ dressed in hospital gown & lying on a hospital bed. Auscultation of mannequin chest will reveal loud harsh breath sounds throughout both lung fields
- ventilator set to 70% inspired oxygen, PEEP 0, ASV mode with MV 100% (or equivalent mode of ventilation)
- pulse oximeter connected to monitor with probe on mannequin
- urinary catheter draining into catheter bag

- 18G & 20G cannulae inserted into left & right anterior cubital fossae
- arterial line inserted into right radial artery
- propofol in 50ml syringe connected to syringe driver infusing at 22ml/hr through left cannula
- 12-lead ECG showing a sinus rhythm (see appendix)
- chest X-ray showing a correctly-positioned endotracheal tube and bilateral pulmonary opacities consistent with severe community-acquired pneumonia (see appendix)
- arterial blood gas (taken on 70% inspired oxygen concentration) showing hypoxaemia, hypercapnia, hypokalaemia & hypomagnesaemia (see appendix)

Phase 2 (set in the fuselage of an aeromedical retrieval plane):
- **i**ntubated female mannequin with #8.0 Portex endotracheal tube in-situ dressed in hospital gown & strapped on to an aeromedical transport stretcher covered by a blanket. The mannequin must be defibrillator safe
- transport ventilator set to 70% inspired oxygen, PEEP 0 (or as set by D in phase 1), ASV mode with MV 100% (or equivalent mode of ventilation)
- transport monitor displaying ECG, arterial & end-tidal carbon dioxide waveforms with audio alarms muted (as would be inaudible in aviation environment)
- pulse oximeter connected to monitor but no trace visible as probe not placed on mannequin (& is hidden from view under the blanket)
- defibrillator (may be part of transport monitor or separate)
- urinary catheter draining into catheter bag
- 18G & 20G cannulae inserted into left & right anterior cubital fossae
- arterial line inserted into right radial artery
- propofol in 50ml syringe connected to syringe driver infusing at 22ml/hr through left cannula

A ‘flight bag’, the contents of which are:
- Crystalloid fluid (3 x 1000ml bags)
- Intravenous giving set
- Amiodarone 150mg vials
- Digoxin 500mcg vials
- Verapamil 5mg vials
- Magnesium Sulphate 10mmol vials
- Potassium Chloride 10mmol vials
- Adrenaline 1mg vials
- Metaraminol 10mg in 10ml pre-mixed syringes
- Noradrenaline 10mg in 100ml pre-mixed bag
- Defibrillator pads (adhesive) with connecting cable

For practical reasons, it would be possible to use the same mannequin for both phases of the scenario provided it can be transferred onto the aeromedical transport stretcher & placed inside the aeroplane fuselage in a timely fashion.

Pre-Scenario Clinical Information:
“You are the medical & nursing members of the Intensive Care aeromedical retrieval team. You have been sent to a small hospital to retrieve a patient who is ventilated in their Emergency Department with a severe pneumonia. You need to bring her back to your ICU as the referring hospital don’t have any ICU beds available.”

All other relevant information will need to be elicited during Phase 1 of the scenario.

Scenario Begins:

Phase 1

Initial Vital Signs:

SET 1

Parameter	Value	Shown On Monitor
ECG trace	Sinus rhythm	Yes
Heart rate	81	Yes
Arterial waveform & blood pressure	132/71	Yes
End-tidal carbon dioxide waveform & value	48	Yes
Respiratory rate	16	Yes
Pulse oximetry	86%	Yes

History:

The retrieval team are met in the Emergency Department by a junior doctor who, in response to direct questioning, is able to provide the following history:

- Lily Blythe is a 76 year old lady who was brought to the Emergency Department earlier today
- she had seen her GP several times in the last week with cough, lethargy & muscle aches & had been treated with antibiotics. Despite this she had continued to get worse & her husband called an ambulance yesterday because she was looking 'a bit blue'
- on arrival to ED she was markedly cyanotic with an increased work of breathing so was rapidly intubated. ICU is full so we have requested transfer to the tertiary hospital
- her past history includes hypertension & chronic obstructive pulmonary disease
- she takes cilazapril & various inhalers
- she has no allergies but beta-blockers make her 'wheezy'
- she had seen her GP for her seasonal influenza vaccination a few months ago & had a 'heart check' which was 'fine'
- she is intubated & ventilated on 70% oxygen. She has an arterial line which is being transduced, 2 peripheral venous cannulae & continuous end-tidal carbon dioxide & ECG monitoring
- she has a heart rate of 81 and a good blood pressure without any vasoactive support
- she was an 'easy intubation'. Other than the suxamethonium given for intubation, she has not had any other paralysing drugs
- if asked any questions about the ventilator, the junior doctor should reply "I don't know anything about ventilators. The anaesthetist set it up before they went home"

On request, D can be shown any of the following (see appendix):

- a post-intubation chest X-ray showing bilateral changes consistent with a severe pneumonia
- an arterial blood gas taken on 70% oxygen showing hypoxaemia,hypercapnia, serum potassium 3.4 mmol/l & serum magnesium 0.6 mmol/l
- a 12-lead ECG showing a sinus rhythm

D is expected to:

- examine the mannequin to ascertain what intravenous & intra-arterial lines are in-situ, check the endotracheal tube position & that the cuff is inflated
- auscultate the chest
- determine the vital signs displayed on the monitor & the current ventilator settings
- check the post-intubation chest X-ray
- notice the PEEP of 0
- notice the hypoxaemia from the saturation probe and either increase the inspired oxygen concentration or increase the PEEP to a higher setting (anywhere between 5 to 15) a few minutes after which the oxygen saturation reading will increase to 94%
- formulate a transfer plan including specific drugs made available (i.e. drawn up) & discuss this with the ICU consultant by telephone. The number of the 'ICU consultant on-call' will be provided which will be the mobile number for one of the simulation team. A transfer plan will only be prompted with 'what's your plan?' if it is not forthcoming from D.

If D fails to perform any of these tasks, N should prompt them to do so.
D may request the administration of a non-depolarising muscle relaxant (such as rocuronium, vecuronium, atracurium or pancuronium). All these are available for administration by N but only if a specific dose is requested.

Phase 1 ends when D has successfully communicated a transfer plan to the ICU consultant on-call & N. The scenario then progresses to Phase 2.

Phase 2

Initial Vital Signs:

SET 2

Parameter	Value	Shown On Monitor
ECG trace	Sinus rhythm	Yes
Heart rate	81	Yes
Arterial waveform & blood pressure	132/71	Yes
End-tidal carbon dioxide waveform & value	49	Yes
Respiratory rate	16 (this must match the rate set on the ventilator as the patient is fully ventilated with no spontaneous breaths)	Yes
Pulse oximetry	91%	No trace or value until probe placed on mannequin

- Begins in-flight at cruise altitude with the flight doctor (D) & flight nurse (N) seated adjacent to the mannequin

- Wait for D to notice absence of saturation trace & reading. Once probe place on finger, show reading of 91%. N can prompt if D fails to notice after some time
- Once the probe is placed, slow desaturation over 3-4 minutes with oximeter value falling to 85%
- D should make an attempt to either increase the inspired oxygen concentration or further increase the PEEP on the ventilator (to an acceptable maximum of 18), or both. Oxygen saturation will rise to a maximum of 89% with these changes
- Occasional ventricular ectopics visible on ECG trace

SET 3

Parameter	Value	Shown On Monitor
ECG trace	Atrial fibrillation	Yes
Heart rate	162	Yes
Arterial waveform & blood pressure	102/67	Yes
End-tidal carbon dioxide waveform & value	38	Yes
Respiratory rate	16	Yes
Pulse oximetry	89%	Yes

- D should recognise the rhythm change with hypotension & communicate this to N
- Appropriate initial treatment could include asking for amiodarone, verapamil or digoxin to be drawn up. Her stated sensitivity to beta-blockers should preclude requesting these
- Before any agents are able to be given, progress to Set 4

SET 4

Parameter	Value	Shown On Monitor
ECG trace	Atrial fibrillation	Yes
Heart rate	181	Yes
Arterial waveform & blood pressure	78/40	Yes
End-tidal carbon dioxide waveform & value	32	Yes
Respiratory rate	16	Yes
Pulse oximetry	82%	Yes

- Appropriate treatment could now include any of the following:
  - attempting to raise the patient’s legs
  - decreasing the propofol infusion rate
  - administering a fluid bolus of 500mls
  - administration of a vasoconstrictor at appropriate dose (0.5 to 1mg of metaraminol)
  - amiodarone (either 150mg or 300mg via slow push)

    - verapamil (5mg neat via slow push over several minutes)
    - digoxin (250 or 500mcg via slow push over several minutes)
    - increasing the inspired oxygen concentration on the ventilator
- Administering any of the above medications will cause a transient decrease in heart rate (to 140) with subsequent rise in blood pressure (to 90 systolic maximum). Patient remains in atrial fibrillation regardless.
- After this patient slowly worsens to vital signs shown in Set 5

SET 5

Parameter	Value	Shown On Monitor
ECG trace	Atrial fibrillation	Yes
Heart rate	192	Yes
Arterial waveform & blood pressure	54/32	Yes
End-tidal carbon dioxide waveform & value	18	Yes
Respiratory rate	16	Yes
Pulse oximetry	74%	Yes

- D must now attempt synchronised defibrillation to convert to sinus rhythm. This involves:
    - communication with both N & the aeromedical crew that the defibrillator needs to be used
    - if asked directly, N will respond with ‘I’ve never used this defibrillator before’
    - finding & correctly placing the adhesive pads onto the mannequin & connecting them to the defibrillator
    - selecting ‘Sync’ mode
        - charging to 100J minimum (biphasic)
        - performing appropriate safety checks prior to shock delivery (communication with team, ensuring no-one is touching patient etc.)
    - if shock <100J selected, patient will remain in AF until a repeat shock at a higher value is delivered
    - if ‘Sync’ mode is **not** selected prior to shock delivery, the patient will convert to ventricular fibrillation, lose all cardiac output & require immediate further unsynchronised defibrillation. If this is not immediately recognised or a second shock is not delivered or D attempts to defibrillate VF with a synchronised shock, the patient will remain in VF. If shock is unsuccessful, D or N should begin CPR for 2 minutes before delivering another (unsynchronised) shock
- If D asks to speak to the ICU Consultant at any point, N should reply that the satellite phone is unable to connect
- Once the patient has been successfully defibrillated (either synchronised from AF or unsynchronised from VF), the patient returns to sinus rhythm, becomes more stable & progresses to vital signs in Set 6

SET 6	Parameter	Value	Shown On Monitor
	ECG trace	Sinus rhythm	Yes
	Heart rate	88	Yes
	Arterial waveform & blood pressure	108/65	Yes
	End-tidal carbon dioxide waveform & value	54	Yes
	Respiratory rate	16	Yes
	Pulse oximetry	92%	Yes

- D may now consider administration of amiodarone 300mg if not already administered

Scenario Ends

Debrief Points:
- How did D & N work as a team?
- Were tasks delegated appropriately?
- Did communication between all team members work well?
- How quickly was the decision to defibrillate made?
- How familiar were you with the pads & defibrillator?
- Could you find everything you needed easily?
- How did the aeromedical environment affect your management?

Clinical Points:
- Management of atrial fibrillation in an unstable elderly patient including discussion around when to use drugs vs progression to defibrillation (especially in context of sedated intubated patient)
- Need for synchronised defibrillation (in AF) vs non-synchronised defibrillation (in VF)
- Appropriate drugs to use & doses to administer
- Management of the unstable patient without central venous access
- End-tidal carbon dioxide as a proxy for cardiac output monitoring

Scenario written by Dr.Alex Psirides, Intensivist, Wellington Regional Hospital.
Conact: **alex.psirides@ccdhb.org.nz**
(adapted from a scenario developed by Dr.Lizzie Speirs, Simulation Fellow, Wellington Hospital)

APPENDICES:
- 12-lead ECG
- Arterial Blood Gas result
- Chest X-ray

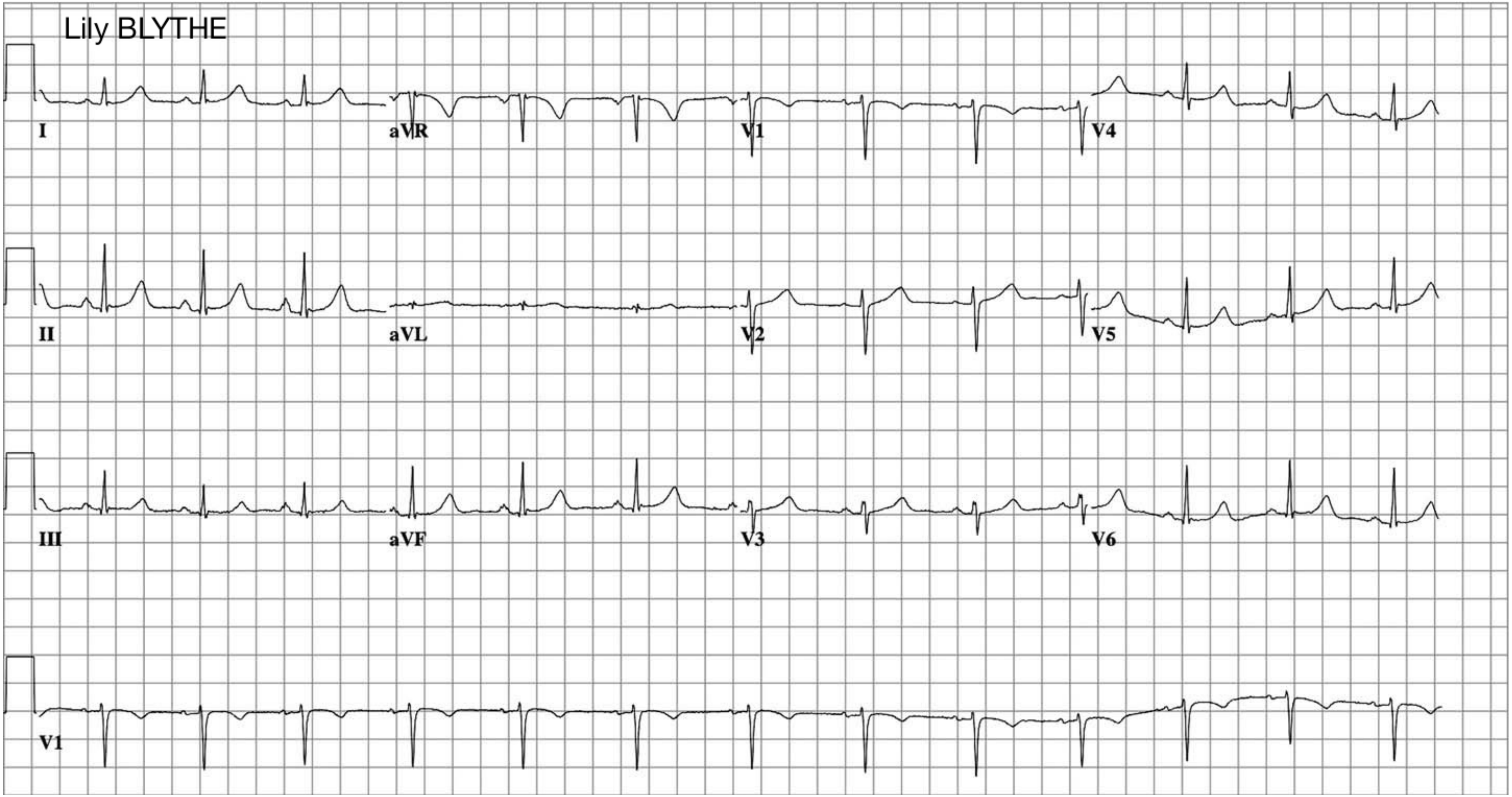
Lily BLYTHE
I
aVR
V1
V4
II
aVL
V2
V5
III
aVF
V3
V6
V1

RADIOMETER ABL800 FLEX

ABL815 ED 08:14
PATIENT REPORT Syringe - S 195uL Sample # 133565

Identifications

Patient ID	QUZ1742
Patient Last Name	BLYTHE
Name checked?	YES
Patient First Name	LILY
Date of birth	17/06/1938
Sample type	Arterial
Inspired Oxygen %	0.70
Operator	Nick Riviera

Acid Base Status

↓pH	**7.233**		[7.350 - 7.450]
↑pCO_2	**52**	mmHg	[35.0 - 45.0]
↓pO_2	**56**	mmHg	[80.0 - 100]
↓$cHCO_3^-$	**18.0**	mmol/L	[20.0 - 30.0]
ABE_c	**-2.3**	mmol/L	[-]

Oximetry Values

ctHb	**118**	g/L	[-]
Hct_c	**37.7**	%	
↓sO_2	**86.1**	%	[95.0 - 98.0]
$p50_e$	**22.9**	mmHg	[-]
↓FO_2Hb_e	**84.9**	%	[94.0 - 97.0]
$FHHb_e$	**3.2**	%	[-]

Electrolyte Values

↓cNa^+	**128**	mmol/L	[139 - 146]
↓cK^+	**3.3**	mmol/L	[3.5 - 4.9]
cCa^{2+}	**1.23**	mmol/L	[1.14 - 1.30]
↓cMg^{2+}	**0.63**	mmol/L	[0.76 - 0.99]

Metabolite Values

↑cGlu	**9.9**	mmol/L	[3.9 - 5.7]

Notes

↑ Value(s) above reference range
↓ Value(s) below reference range
c Calculated value(s)
e Estimated value(s)

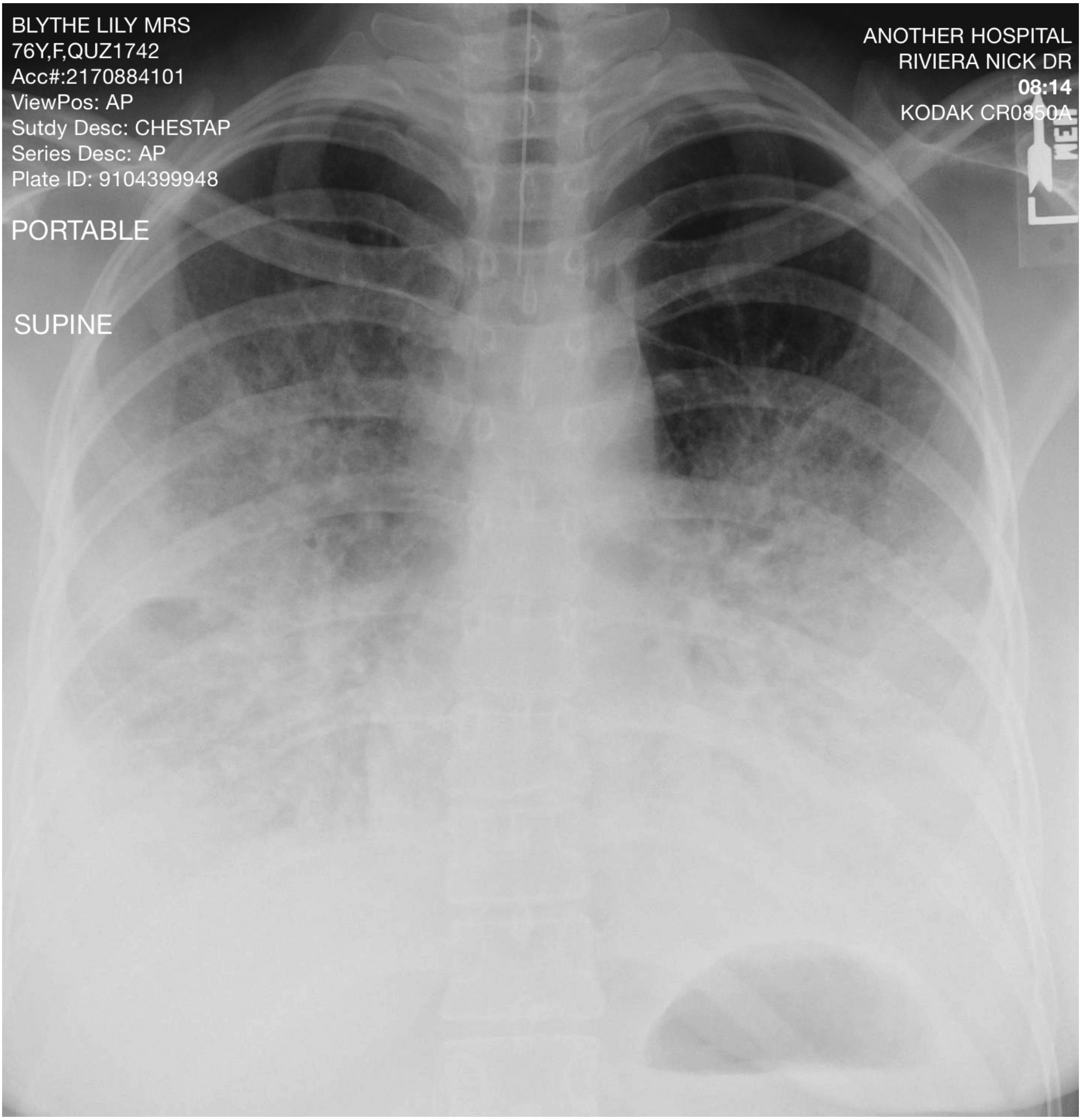

Reference for management of tachycardias: **https://www.resus.org.uk/pages/tachpost.pdf**

Aeromedical Simulation Standardised Pre-Briefing

You are the medical & nursing members of an Intensive Care aeromedical retrieval team. You have been sent to a small hospital to retrieve a patient who is ventilated in the Emergency Department with a severe pneumonia. You have been asked to transfer her to your ICU as the referring hospital don't have any ICU beds available.

The scenario occurs over 2 phases set in different areas. The first occurs in the resuscitation bay of an emergency department, the second in the back of the retrieval aircraft flying at cruise altitude. You will be moved to the area for phase 2 when phase 1 has concluded.

The monitoring equipment, ventilator & medication you have available to you are the same as those you would have access to during an aeromedical retrieval.

The mannequin should be treated as if it were a real patient. You are able to monitor, cannulate, intubate, ventilate, defibrillate & auscultate it just as you would in real life.

Phase 1 now begins, immediately after your arrival at the small hospital. A very junior doctor is here and can answer some questions that you may have about the patient.
