# Supplementary material for: Non-technical skills evaluation in the critical care air ambulance environment: introduction of an adapted rating instrument - an observational study
Source: Scand J Trauma Resusc Emerg Med. 2016 Mar 8;24:24. doi: 10.1186/s13049-016-0216-5 (PMC4784461; doi:10.1186/s13049-016-0216-5)
Supplement: Additional file 3: — Aeromedical non-technical skills assessment form and rating scale. (PDF 266 kb) [file 13049_2016_216_MOESM3_ESM.pdf]

Aeromedical non-technical skills: Assessment form and rating scale

Name/Study ID: **Observer/rater:** **Date:**

**Categories**	*Category rating (1 – 5)	**Elements**	*Element rating (1 – 5)	**Notes**
Task Management		Planning and preparing		
		Prioritising		
		Maintaining standards		
		Identifying and utilising resources		
Team Working		Coordinating activities with the team		
		Exchanging Information		
		Using authority & assertiveness		
		Assessing capabilities		
		Supporting others		
Situation awareness		Gathering information		
		Recognising & understanding		
		Anticipating		
Decision making		Identifying options		
		Balancing risks & selecting options		
		Re-evaluating		

* 1 = Poor; 2 = Marginal; 3 = Acceptable; 4 = Good; 5 = Excellent; N = skill was not required in this scenario

The clinician is rated based on what is expected for a trained and competent aeromedical clinician. Round down to the next lower integer rating if you feel performance was best described in between two scale points

GLOBAL RATING: (Mark with a circle): **Poor 1 – 2 – 3 – 4 – 5 – 6 – 7 Excellent**

Rating Options	Descriptor
5 – Excellent<br>*Exceptional*	Extremely good performance which could serve as a model example for others; patient safety enhanced
4 – Good<br>*Strong*	A consistently high standard of performance, patient safety assured
3 – Acceptable<br>*Adequate*	Satisfactory performance but could be improved; patient safety not affected
2 – Marginal<br>*Borderline*	Performance gives rise to concern; patient safety potentially compromised
1 – Poor<br>*Obviously unacceptable*	Absence of behaviour required by the situation; performance endangered or potentially endangered patient safety

* Round down to the next lower integer rating if you feel performance was best described in between two scale points
