# Supplementary material for: Non-technical skills evaluation in the critical care air ambulance environment: introduction of an adapted rating instrument - an observational study
Source: Scand J Trauma Resusc Emerg Med. 2016 Mar 8;24:24. doi: 10.1186/s13049-016-0216-5 (PMC4784461; doi:10.1186/s13049-016-0216-5)
Supplement: Additional file 4: — Behavioural descriptor modifications based on content evaluation survey. (PDF 259 kb) [file 13049_2016_216_MOESM4_ESM.pdf]

Behavioural descriptor modifications following content validity survey

NTS main category and skill element	Original behavioural descriptor	Revised behavioural descriptor
Task Management: Providing and maintaining standards	*Follows established protocols and guidelines eg. Reviews checklists before key time-points in the transfer CVI = 0.72*	Follows established protocols and guidelines when appropriate
Team working: Exchanging information	*Avoids criticising CVI = 0.61*	Expresses concerns constructively
Team working: Assessing capabilities	*Asks new team member about their experience CVI = 0.72*	Clarifies the experience of team members they have not worked with before
Situational awareness: Gathering information	*Conducts frequent scan of the environment, CVI = 0.72*	Conducts a frequent scan of the clinical and aviation environment
Decision making: Identifying options	*Seeks input on various transport related issues with patient or family, CVI = 0.64*	Seeks input on various transport related issues with all relevant parties
The content validity index (CVI) is calculated by the number of respondents who rated the importance of a behaviour as 'very important' or 'essential', divided by the total number of respondents. A CVI of less than 0.75 is considered to potentially indicate low content validity [21].		
